# Interpretation of Parathyroid Hormone Levels in Renal Impairment

**DOI:** 10.7759/cureus.25819

**Published:** 2022-06-10

**Authors:** Ifeanyi F Nwosu, Cece E Ibeson, Adedoyin Olawoye, Htin Kyaw, Kelash Kumar, Celestine Odigwe, Chukwunonso A Nwosu, Anthony Oshunsanya

**Affiliations:** 1 Internal Medicine, Maimonides Medical Center, Brooklyn, USA; 2 Internal Medicine, Thomas Hospital, Fairhope, USA; 3 Epidemiology and Public Health, Kaonic Research, Gaithersburg, USA

**Keywords:** urinary casts, urinalysis, chronic kidney injury, acute kidney injury, parathyroid hormone

## Abstract

Distinguishing between acute kidney injury and chronic kidney disease (CKD) in an emergency setting may pose a conundrum for physicians, especially when a patient’s medical history and records are unknown. Parathyroid hormone (PTH) has proved valuable as a marker of CKD and is frequently assayed for this reason. The use of PTH as a sole marker of CKD may be misleading in certain conditions, and for this reason, physicians need to interpret PTH values with caution. In patients with no existing medical records, it is vital to consider their overall clinical picture, an accurate interpretation of urinalysis and urine microscopy, and the PTH values when making the initial management decisions.

## Introduction

Hyperparathyroidism is a potential cause of kidney failure, and an elevated parathyroid hormone (PTH) level is a marker of kidney impairment [1,2]. Deranged kidney function is a common reason for emergency room presentation and acute medical admissions. An immediate challenge facing physicians in an emergency setting is distinguishing acute kidney injury (AKI) from chronic kidney disease (CKD) in patients with no existing medical records for comparison. Determining the acuity of kidney impairment is relevant as it guides the initial management and aggressiveness of fluid resuscitation, among other treatments.

PTH is a commonly used marker for assessing kidney impairment, and elevated PTH levels are mostly associated with CKD [2]. However, sporadic cases of markedly elevated PTH have been reported in the literature in patients with AKI with no evidence of parathyroid gland disorders [3,4]. Here, we report a case of acutely elevated PTH in a patient presenting with AKI with complete normalization of PTH and renal function following fluid management within 48 hours. We aim to assess the role of PTH as an acute-phase reactant and highlight the need to interpret PTH values with caution when managing patients with renal impairment.

## Case presentation

A 34-year-old man with a medical history of alcohol misuse disorder and human immunodeficiency virus infection on cabotegravir-rilpivirine combination presented to the emergency department (ED) following a witnessed seizure-like activity. He had a brief episode of a generalized tonic-clonic seizure lasting for less than a minute and was associated with post-ictal confusion. The patient was lethargic for about a week, and his last alcohol intake was a week before the presentation. He engaged in binge drinking for over two weeks and would drink approximately a bottle of vodka daily. He denied any fever, headache, neck pain, visual symptoms, hallucinations, cough, abdominal pain, or urinary symptoms but endorsed having nausea and generalized weakness without vomiting. He was compliant with his medication and regular with his follow-ups.

In the ED, his initial vital signs were a temperature of 37.2°C, blood pressure of 92/50 mmHg, mean arterial pressure of 64 mmHg, heart rate of 116 beats per minute, respiratory rate of 20 cycles per minute, and oxygen saturation of 100% on room air. The patient was lethargic but well oriented with a Glasgow coma scale score of 15 and no focal neurological deficits. The rest of the systemic examinations were normal. Investigations in the ED showed dyselectrolytemia with severe hyponatremia, significantly elevated creatinine and PTH, and abnormal urinalysis with coarse granular casts, as shown in Table 1.

**Table 1:** Pertinent initial laboratory results. eGFR: estimated glomerular filtration rate; HPF: high-power field; LPF: low-power field; PCR: polymerase chain reaction

Biochemistry/Microscopy	Result	Reference range
Sodium (mmol/L)	111	135–149
Potassium (mmol/L)	2.5	3.4–4.8
Urea (mg/dL)	61.7	7–21
Creatinine (mg/dL)	4.2	0.3–1.1
eGFR (mL/minute/1.73 m^2^)	21	>60
Calcium (mg/dL)	9.6	8.2–10.1
Phosphorus (mg/dL)	3.4	2.2–5.5
Parathyroid hormone (pg/mL)	204	24–85
Anion gap (mmol/L)	25	3–11
Creatine phosphokinase (IU/L)	412	59–367
Serum osmolarity (mOsm/kg)	266	275–295
Lactate (mmol/L)	2.8	0.5–1.6
pH	7.46	7.35–7.45
Beta-hydroxybutyrate (mmol/L)	3.38	0.02–0.27
Random blood glucose (mg/dL)	204	59–140
Hemoglobin A1C (%)	5.6	4–6
Alcohol level (mg/dL)	<10	<10
25-hydroxy vitamin D (ng/mL)	43	20–50
HIV RNA PCR (copies/mL)	Uundetectable	Negative
CD4 (/µL)	144	489–1,457
Urine red blood cells (/HPF)	5–10	0
Urine white blood cells (/HPF)	2	<5
Urine coarse granular casts (/LPF)	10–25	
Urine hyaline casts (/LPF)	2–5	
Urinary sodium (mmol/L)	11	<20
Urine osmolarity (mOsm/kg)	411	500–800

He had a normal blood count and vitamin D level, negative urine toxicology, and computed tomography (CT) of the head was negative for any acute infarction, intracranial hemorrhage, or mass lesion. He was initially resuscitated with 2 L of lactated Ringer’s solution while in the ED but was subsequently placed on fluid restriction for a gradual correction of his hyponatremia. His potassium was adequately repleted. On day one of admission to the medical intensive care unit, the patient’s renal function had improved remarkably with normalization of creatinine to 1.2 mg/dL, blood urea of 31.2 mg/dL, and estimated glomerular filtration rate (eGFR) of >60. A repeat PTH on day two showed normal levels of 71 pg/mL. The kidney function subsequently continued to downtrend.

## Discussion

PTH is a polypeptide protein secreted by the parathyroid glands [5]. It plays a crucial role in calcium and phosphate homeostasis through its effects on the bone, renal tubules, and gastrointestinal tract [5]. In CKD, there is impaired renal vitamin D synthesis where PTH plays a major role in activating the enzyme 1-alpha-hydroxylase for the hydroxylation of 25-hydroxyvitamin D to 1,25-hydroxyvitamin D [6]. Over time, the ensuing vitamin D deficiency results in reduced gastrointestinal absorption of calcium, positive feedback to the parathyroid glands, parathyroid hyperplasia, and secondary hyperparathyroidism [6]. In late CKD, PTH is unable to ensure renal phosphate excretion, and the net effect of CKD and secondary hyperparathyroidism are hypocalcemia, hyperphosphatemia, and low vitamin D levels [7-9]. Consequently, calcium and vitamin D supplementation and phosphate binders are initiated in late CKD and dialysis patients [10].

At presentation, our patient had normal calcium, phosphate, and vitamin D levels. The hyperparathyroidism was transient and likely secondary to AKI. This is evidenced by the complete reversal of the PTH within 48 hours of admission and treatment. Furthermore, the short-lived rise in serum PTH level is supported by the normal serum calcium, phosphate, and vitamin D levels. In a patient with primary hyperparathyroidism as the cause of elevated PTH, serum PTH is unlikely to normalize with fluid hydration. However, the calcium level may reduce if there is co-existing hypercalcemia. Conversely, in secondary hyperparathyroidism from CKD, one would expect abnormally elevated serum phosphate, hypocalcemia, and low vitamin D levels. This was not the case with our patient. In a double-blind, randomized control trial by Zaloga and Teres, PTH was found to be elevated in critically ill patients in the absence of kidney disease, suggesting that PTH may be a stress hormone [11]. Similarly, Czarnik et al. observed elevated PTH levels among hemodialysis patients during an acute illness with sepsis and a reversal in a stable state [12].

A simple urinalysis and microscopy can provide valuable information. Whereas waxy casts are seen in CKD, the findings of coarse granular casts, characteristic of acute tubular necrosis AKI should prompt the suspicion of AKI [13]. A hyaline cast that can be seen in normal healthy patients and is mostly non-specific [14] was seen in our patient together with coarse granular casts. Although studies overwhelmingly favor elevated PTH in patients with CKD, physicians should be aware that elevated PTH can follow AKI. Therefore, a combination of the patient’s medical history, laboratory parameters, and overall clinical presentation is needed when deciding to aggressively fluid resuscitate a patient with no accessible prior laboratory values presenting with an abnormal kidney function.

## Conclusions

The use of PTH as a sole marker of CKD in patients presenting with an acute illness without prior history of CKD, or baseline creatinine, should be avoided. Interpreting PTH levels with another adjunct test, including urinalysis and microscopy, will help improve the accuracy of diagnosis to help in immediate patient management.
